# Effects of oxiracetam combined with ginkgo biloba extract in the treatment of acute intracerebral hemorrhage: A clinical study

**DOI:** 10.1002/brb3.1661

**Published:** 2020-06-12

**Authors:** Xiu‐Xiu Li, Shi‐Hui Liu, Su‐Jing Zhuang, Shi‐Feng Guo, Shou‐Liang Pang

**Affiliations:** ^1^ Department of Neurology Linyi Central Hospital Linyi China

**Keywords:** acute intracerebral hemorrhage, ginkgo biloba extract, oxiracetam, routine treatment

## Abstract

**Purpose:**

The present clinical study was conducted to investigate the effect of oxiracetam combined with ginkgo biloba extract in treating patients with acute intracerebral hemorrhage.

**Methods:**

Ninety‐eight patients with acute cerebral hemorrhage admitted to our hospital were divided into three groups. The differences of brain edema and cerebral hemorrhage were compared between the three groups after 1 and 2 weeks of treatment, and the recovery of neurological function, serum inflammatory factors, AQP‐4, MMP‐9, cognitive function, activities of daily living, and adverse reactions were compared between the three groups after 2 weeks of treatment.

**Results:**

There was no significant difference among the three groups before treatment (*p* > .05). After treatment, the recovery of neurological function, serum inflammatory factors, AQP‐4, MMP‐9 levels, cognitive function, and activities of daily living were improved. Among them, the neurological function recovery, serum inflammatory factors, AQP‐4, MMP‐9 levels, cognitive function, and activities of daily living in the combined treatment group and the control group elicited greater results than those in the routine group. The results of the combined treatment group showed the most significant difference (*p* < .05). The concentration of IL‐6 decreased from 135.98 ± 12.54 to 91.83 ± 7.69 pg/ml, AQP‐4 from 227.55 μg/L ± 21.06 to 114.31 ± 9.22 μg/L, and MMP‐9 from 172.39 ± 9.81 to 94.98 ± 5.01 ng/ml. In addition, the neurological function recovery, the levels of serum inflammatory factors, cognitive function, and activities of daily living in the combined treatment group were better than those in the control group (*p* < .05). The mean score of MRS in the combined treatment group decreased from 3.36 ± 0.98 at admission to 1.91 ± 0.38.

**Conclusion:**

Oxiracetam combined with Ginkgo biloba extract in the treatment of acute cerebral hemorrhage has a significant improvement effect.

## Graphical abstract

The combination of oxiracetam and ginkgo biloba extract in patients with acute intracerebral hemorrhage has potential in term of significantly improving the therapeutic effect and alleviating neurological deficit, reducing the absorption of hematoma and edema, and it is safe and beneficial to the rehabilitation of patients.

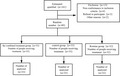

## 1 INTRODUCTION

Acute intracerebral hemorrhage is a life‐threatening condition of acute cerebrovascular disease, which is a primary and nontraumatic intracerebral hemorrhage. Its main characteristics include high morbidity, acute onset, high recurrence rate, high disability rate, and mortality. Patients who survive the attack of acute intracerebral hemorrhage may develop varying degrees of sequelae that often have a serious impact on the quality of life and mental health of the patients (Krel et al., 2019; Wang, Zhang, You, Zhu, & Zhou, 2019). At present, drug treatment, minimally invasive hematoma aspiration, and surgical intervention are often used to treat the disease, but the effect has some limitations, especially to restore the neurological function of patients (Kakita et al., 2019). Among them, the surgical intervention is usually specific for patients with moderate disturbance of consciousness at the time of admission. After the occurrence of the disease, intracranial hematoma will cause compression to the surrounding normal brain tissue to develop secondary brain injury; at the same time, perihematoma will reduce cerebral blood flow and cause secondary brain injury. Moreover, the principle of conservative medical treatment includes the reduction of blood pressure, hemostasis, and prevention of complications (Chen, Ma, et al., 2019). Therefore, the patients with early acute cerebral hemorrhage should seek early effective interventions. Oxiracetam is a new type of neurotrophic drug, which can accelerate the energy metabolism of brain tissue and improve the utilization of glucose by activating glycolysis. Ginkgo biloba extract has many biological effects, which can improve the blood flow and microcirculation of the whole brain, prevent hypoxia damage, improve tissue metabolism, and reduce capillary permeability. However, the therapeutic effect of Ginkgo biloba extract combined with oxiracetam on acute cerebral hemorrhage has not been studied. In this study, three treatment methods were used to compare the effects of Ginkgo biloba extract and oxiracetam on the recovery of neurological function in patients with acute cerebral hemorrhage.

## 2 MATERIALS AND METHODS

### 2.1 General information

**FIGURE 1:**
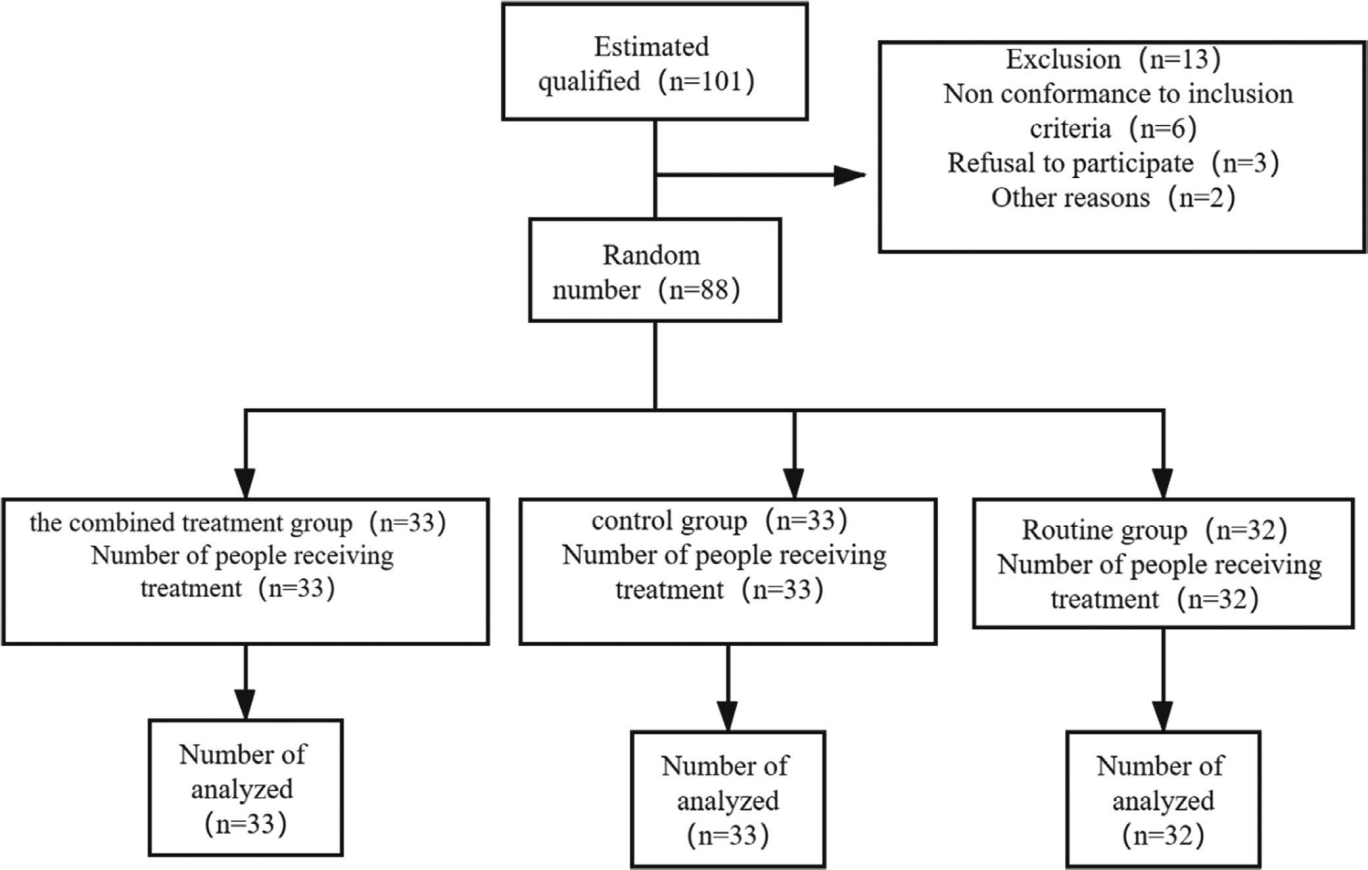
The experiment Consort diagram. The estimated number of people in this experiment is 101, which is not included in the inclusion criteria, or patients refuse to participate in this experiment or for some other reasons. The total number of people who finally participate in this experiment is 88. There were 33 patients in the combined treatment group, and all of them participated in the combined treatment; there were 33 patients in the control group and 32 in the conventional group

A total of 88 patients who had suffered from acute intracerebral hemorrhage were admitted to the Department of Neurology of our hospital from January 2018 to June 2019. According to the age of admission and the life characteristics of the patients, the patients were divided into three groups. There was no significant difference in the detection of admission. They were the combined treatment group, the control group, and the routine group. The three groups were given different treatment plans. In the combined treatment group, there were 18 men and 15 women, aged from 27 to 68 years, with an average age of 49.08 ± 6.97 years. In the control group, there were 19 men and 14 women, aged from 28 to 67 years, with an average age of 50.87 ± 6.81 years. In the routine group, there were 32 cases including 18 men and 14 women, aged from 27 to 69 years old, with an average age of 51.09 ± 7.01 years. There was no significant difference in age and other general data among the three groups (*p* > .05). All patients signed informed consent forms and volunteered to participate in this study. The clinical data of the patients are shown in Table 1 in details. This experiment Consort diagram is shown in Figure 1.

**TABLE 1:** General data of three groups of patients

Parameter	Combined treatment group (*n* = 33)	Control group (*n* = 33)	Routine group (*n* = 32)	*x* ^2^/*t*	*p* value
Gender (case, %)					
Male	18	19	18	3.471	.804
Female	15	14	14		>.05
Age ( $\bar{x}\pm s$ , year)	49.08 ± 6.97	50.87 ± 6.81	51.09 ± 7.01	1.971	.428
Time from symptom onset to admission ( $\bar{x}\pm s$ , hr)	5.73 ± 1.01	5.64 ± 1.09	5.74 ± 1.09	2.183	.916
GCS ( $\bar{x}\pm s$ , score)	10.75 ± 1.98	10.73 ± 1.89	10.81 ± 1.92	3.098	.985
Hypertension (case, %)	13 (39.40)	14 (42.42)	13 (40.63)	1.632	.838
Coronary artery disease (case, %)	10 (30.30)	9 (27.27)	10 (31.25)	2.765	.972
Diabetes (case, %)	8 (24.24)	10 (30.30)	9 (28.13)	3.382	.51
Hyperlipidemia (case, %)	8 (24.24)	7 (21.21)	8 (25.00)		.769
Site of bleeding (case, %)					
Basal ganglia region	12 (36.36)	14 (42.42)	13 (40.63)	3.971	.621
Cerebellum	8 (24.24)	9 (27.27)	9 (28.13)	3.765	.715
Lobes	8 (24.24)	7 (21.21)	6 (18.75)	3.814	.629
Brainstem	5 (15.15)	3 (9.09)	4 (12.50)	2.312	.532
Bleeding volume ( $\bar{x}\pm s$ , ml)	21.62 ± 1.41	21.65 ± 1.39	21.59 ± 1.31	1.348	.984

Note

Time from symptom onset to admission (hr), hr represents hour.

### 2.2 Selection criteria

#### 2.2.1 Inclusion criteria

(a) All patients met the diagnostic criteria of Chinese guidelines for the diagnosis and treatment of acute ischemic stroke in 2010 (Wang, Fang, Yu, & Li, 2018) and were confirmed by cranial CT and/or MRI, and the time from onset to admission was <12 hr; (b) the amount of bleeding was <30 ml, and the hemorrhage did not cause the enlargement of the ventricular system; (c) the vital signs of patients were stable, with conscious or lethargic condition; (d) patients had no other serious organic diseases; (e) there were no diseases such as heart, brain, and kidney diseases before cerebral hemorrhage; (f) the Glasgow Coma Scale (GCS) of at admission was >8; and (g) there was no indication of operation. The current study was approved by the hospital ethics committee, and all of patients signed the informed consent form.

#### 2.2.2 Exclusion criteria

The characteristics of exclusion criteria are as follows: (a) Patients with moderate‐to‐severe coma (GCS score ≤8) (Brennan, Murray, & Teasdale, 2018) or cerebral hernia formation; (b) patients with traumatic cerebral hemorrhage and other causes of intracranial hemorrhage; (c) patients with bleeding volume >30 ml who needed surgical intervention; (d) patients were complicated with serious heart, lung, liver, kidney, and other important organ diseases; (e) patients with aphasia; (f) patients with massive cerebral hemorrhage, or in a coma; (g) patients who died 72 hr after admission; (h) patients were accompanied by other serious organic diseases; (i) patients receiving surgical treatment; (j) incomplete clinical data; and (k) patients were allergy to drugs of this study.

### 2.3 Treatment

The routine treatment scheme is as follows: closely observe the four vital signs and consciousness of patients, actively control brain edema, reduce intracranial pressure; 1‐hr systolic blood pressure is higher than 140 mmHg, mannitol of 1–4 g/kg.day is required to reduce blood pressure, patients with systolic blood pressure greater than 220 mmHg are given continuous intravenous medication to strengthen blood pressure reduction, and esmolol hydrochloride is used; the epilepsy caused by cerebral hemorrhage is focal epilepsy, which can be distinguished as conscious and impaired consciousness, and some patients with high fever can cause epilepsy, so patients will be treated with antiepileptic therapy; real‐time monitoring of blood glucose, regardless of the patient's history of diabetes, to ensure that it is within the normal range; nutritional support for patients 24–48 hr after the onset of the disease; the use of neuroprotective agents to give neuroprotection to patients; and anti‐infection for all patients, in addition to ensuring that patients breathe smoothly, timely removal of respiratory secretions. The main purpose was to relieve vasospasm and regulate microcirculation. The patients in acute stage were given early rehabilitation, and the complications such as intracranial rebleeding, stress ulcer, and respiratory system infection were prevented and treated. Patients in control group were given the above treatment and 6‐g intravenous oxiracetam injection (trade name: oxiracetam injection; specification: 5 ml: 1.0 g; manufacturer: Fujian Haoanxin Pharmaceutical Co., Ltd.; No.: H20183017) that was dissolved into normal saline 250 ml once a day for 2 weeks. In the combined treatment group, 17.5‐g Ginkgo biloba extract injection was given on the basis of the treatment in the control group (trade name: ginkgo biloba extract injection; specification: 5 ml: 17.5 mg*10 branch; manufacturer: Taiwan Jisheng Chemical Pharmaceutical Co., Ltd.; No.: HC20140019) that was dissolved into normal saline 500 ml, once a day, for 2 weeks.

### 2.4 Observation indicators

After 2 weeks of administration, hematoma, edema absorption, neurological function recovery, serum inflammatory factors, AQP‐4, MMP‐9, cognitive function, activities of daily life, and adverse reactions were compared among the three groups.

#### 2.4.1 The absorption cerebral edema and hematoma

The volume of cerebral hematoma and cerebral edema shown by craniocerebral CT was quantitatively analyzed by Image Pro Plus image analysis system before treatment, 1 week and 2 weeks after treatment. The area of the low‐density zone around the cerebral hematoma and the cerebral edema was measured by the system, multiplied by the height of the layer, and the absolute brain edema volume at each time point = the low‐density zone volume measured by CT‐the original brain hematoma volume, which was the volume of cerebral edema and the volume of cerebral hematoma at different time points in each patient.

#### 2.4.2 Neurological function recovery

Before treatment and 2 weeks after treatment, modified Rankin Scale (mRS) (Dewilde et al., 2017) was used to evaluate the neurological function recovery status of patients. All evaluators need to be formally trained to administer this scale, and they are unaware of the treatment group for each subject. The score was 0–6. High score represented bad neurological function recovery.

#### 2.4.3 Serum levels of inflammatory factors, AQP‐4, and MMP‐9

Fasting venous blood was collected before and 2 weeks after treatment and stored after centrifugation. Interleukin‐6 (IL‐6), aquaporin 4 (AQP‐4), matrix metalloproteinase‐9 (MMP‐9), hypersensitive C‐reactive protein (hs‐CRP), and tumor necrosis factor‐α (TNF‐α) were quantitatively detected by enzyme‐linked immunosorbent assay kit produced by R&D Systems Co., Ltd. (purchased from Shanghai Crystal Anti‐Bioengineering Co., Ltd.) and DNM‐9606 automatic enzyme labeling instrument produced by Pulang in Beijing.

#### 2.4.4 Cognitive function

The cognitive function of all patients was evaluated by Montreal Cognitive Evaluation scale (MoCA) ( Borland et al., 2017) before and 2 weeks after treatment, including a total of eight items: visual space executive function (0–5), orientation (0–6), delayed recall (0–5), abstract thinking (0–2), memory (no score), speech function (0–3), Naming (0–3), and attention and concentration (0–6). The full score was 30 points, and the cognitive function was normal = score ≥26 points.

#### 2.4.5 Activities of daily living (ADL)

Activities of daily living (ADL) scale (Liu, Li, Chen, Liu, & Cai, 2018) was used to evaluate the activities of daily living before and 2 weeks after treatment, including a total of 10 items: eating, dressing, defecation, urination, going up and down stairs, going to the toilet, bathing, transferring, modification, and activity. The lowest score was 10 points, and the highest score was 10 points. High score represented good ability of daily living.

#### 2.4.6 Adverse reactions

Dizziness, nausea, vomiting, and diarrhea during treatment were recorded.

### 2.5 Statistical analysis

In the current study, the data were analyzed by SPSS20.0 software, using $\bar{x}\pm s$ and *t* test to represent the measurement data of neurological function and cerebral hemodynamics. The case number and percentage was used to express the clinical curative effect. In addition, *t* test was used to analyze the indexes before and after treatment. *χ* ^2^ test was used to compare the differences of gender, hypertension, and other components in different groups and was used to compare the overall efficiency of the two therapies. A *p* < .05 represented significant statistical difference.

## 3 RESULTS

### 3.1 Comparison of cerebral edema and cerebral hematoma among the three groups

**FIGURE 2:**
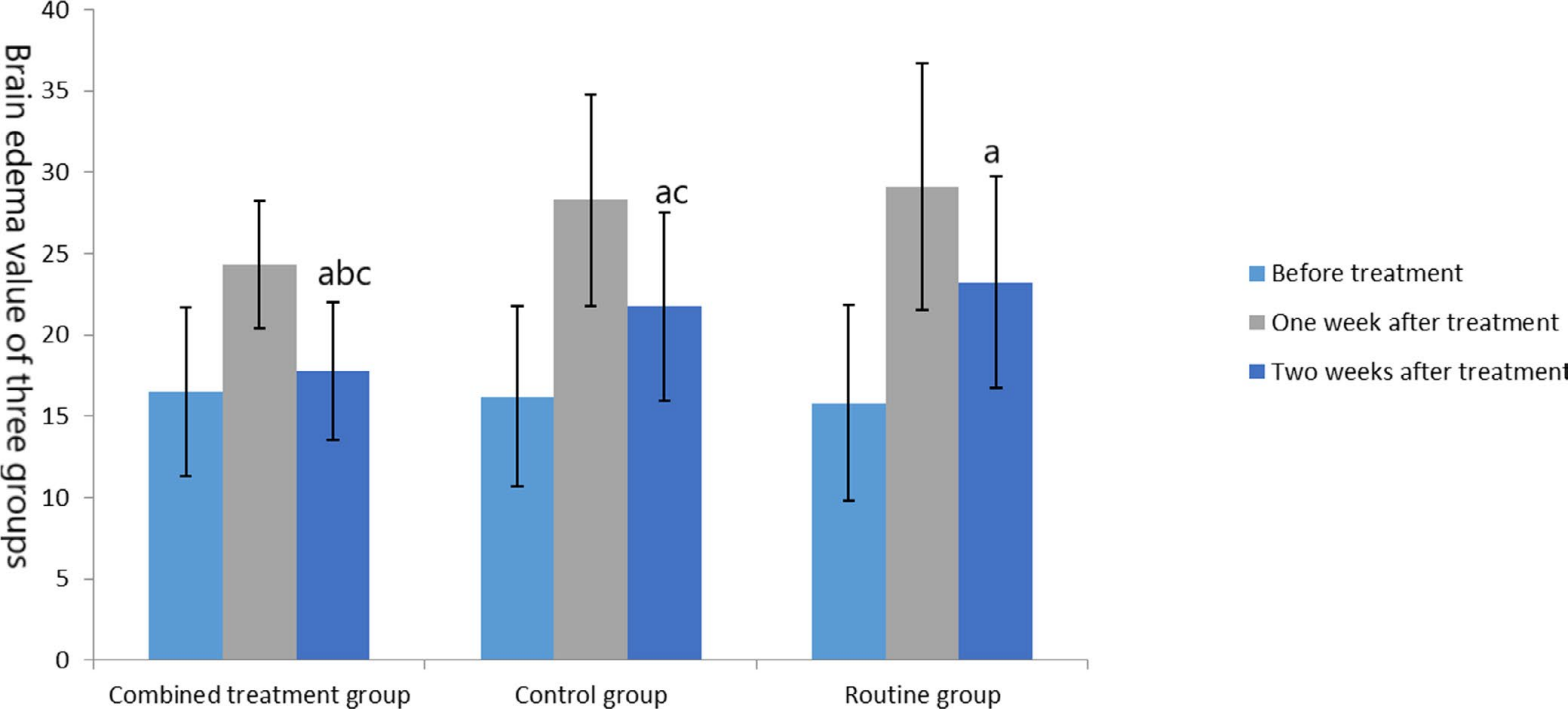
Comparison of brain edema in the three groups. The data of the combined treatment group, the control group, and the conventional group before treatment were 16.5 ± 5.19, 16.21 ± 5.55, and 15.8 ± 6.01 ml, respectively. The data after 1 week treatment were 24.31 ± 3.88, 28.27 ± 6.47, and 29.10 ± 7.59 ml, respectively. The data after 2 weeks treatment were 17.79 ± 4.21, 21.74 ± 5.77, and 23.22 ± 6.52 ml, respectively. Before treatment, compared with the same group (^a^ *p* < .05); after treatment, compared with the control group (^b^ *p* < .05) and compared with the routine group (^c^ *p* < .05)

**FIGURE 3:**
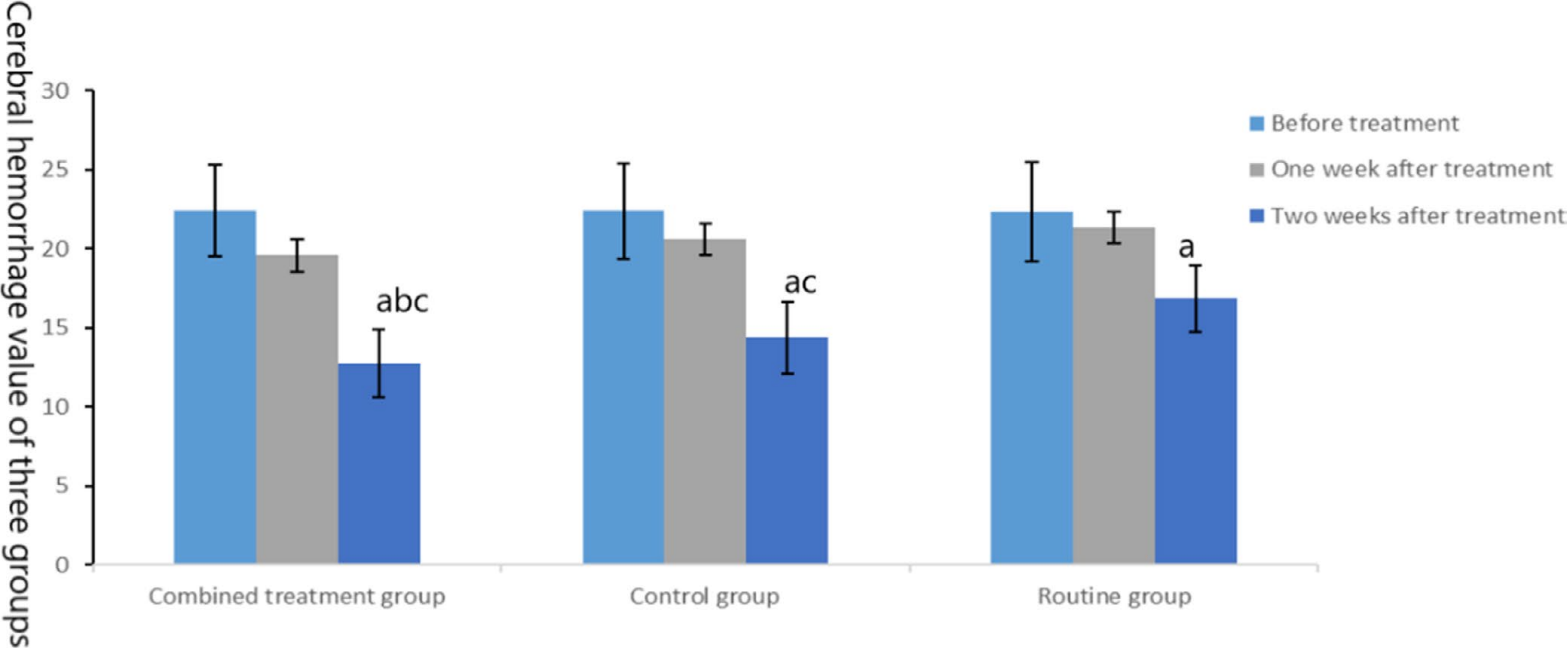
Comparison of cerebral hematoma in three groups. The data of the combined treatment group, the control group, and the conventional group before treatment were 22.41 ± 2.92, 22.38 ± 3.01, and 22.31 ± 3.14, respectively. The data after 1 week treatment were 19.56 ± 2.38, 20.56 ± 0.06, and 21.36 ± 2.29, respectively. The data after 2 weeks treatment were 12.76 ± 2.17, 14.38 ± 2.26, and 16.83 ± 2.09, respectively. Before treatment, compared with the same group (^a^ *p* < .05); after treatment, compared with the control group (^b^ *p* < .05) and compared with the routine group (^c^ *p* < .05)

Before treatment, there was no significant difference in brain edema and hematoma among the three groups (*t* = 0.33, 0.009, *p* = .72, .991). After treatment, outcomes of the three groups were improved, among which the cerebral edema and cerebral hematoma in the combined treatment group and the control group were lower than those in the routine group, and the cerebral edema and hematoma in the combined treatment group were lower than those in the control group (*t* = −3.373, −3.809, −2.413, −2.97, *p* = .001, <.001, .019, .004), as shown in Figures 2 and 3.

### 3.2 Comparison of neurological recovery among the three groups

Before treatment, there was no significant difference in mRS score and case number of mRS > 3 among the three groups. After treatment, outcomes all the three groups were improved, and mRS score and cases of mRS > 3 in the combined treatment group and the control group were better than those in the routine group, and the mRS score and case number of mRS > 3 in the combined treatment group were better than those in the control group (*t* = −3.657, 4.19, *p* = .001, .041×), as shown in Table 2.

**TABLE 2:** Comparison of neurological function recovery among the three groups

Group	mRS (score)		mRS > 3 (case)	
	Before treatment	After treatment	Before treatment	After treatment
Combined treatment group (*n* = 33)	3.36 ± 0.98	1.91 ± 0.38^abc^	27 (81.82)	8 (24.24)^abc^
Control group (*n* = 33)	3.32 ± 1.01	2.32 ± 0.52^ac^	26 (78.79)	12 (36.36)^ac^
Routine group (*n* = 33)	3.33 ± 0.87	2.58 ± 0.47^a^	25 (78.13)	14 (43.75)^a^

Note

Before treatment, compared with the same group (^a^ *p* < .05); after treatment, compared with the control group (^b^ *p* < .05) and compared with the routine group (^c^ *p* < .05).

### 3.3 Comparison of serum inflammatory cytokines, AQP‐4, and MMP‐9 levels among the three groups

Before intervention, there was no significant difference in IL‐6, AQP‐4, MMP‐9, hs‐CRP, and TNF‐α index among the three groups (*t* = 0.115, 0.082, 0.171, 0.158, 0.071; *p* = .891, .921, .843, .854, .932). After treatment, the indexes of IL‐6, AQP‐4, MMP‐9, hs‐CRP, and TNF‐α in the combined treatment group and the control group were better than those in the routine group, and the indexes of CRP, PCT, and WBC in the combined treatment group were better than those in the control group, as shown in Table 3.

**TABLE 3:** Comparison of serum inflammatory cytokines, AQP‐4, and MMP‐9 levels between the three groups ( $\bar{x}\pm s$ )

Group	Time	IL‐6 (pg/ml)	AQP‐4 (μg/L)	MMP‐9 (ng/ml)	hs‐CRP (mg/L)	TNF‐α (ng/L)
Combined treatment group (*n* = 33) Control group (*n* = 33)	Before treatment	135.98 ± 12.54	227.55 ± 21.06	172.39 ± 9.81	5.83 ± 0.83	161.04 ± 11.09
	2 weeks after treatment	91.83 ± 7.69^abc^	114.31 ± 9.22^abc^	94.98 ± 5.01^abc^	2.49 ± 0.18^abc^	101.09 ± 8.93^abc^
Routine group (*n* = 33) Combined treatment group (*n* = 33)	Before treatment	134.62 ± 10.96	226.52 ± 9.05	171.60 ± 9.52	5.91 ± 0.91	160.87 ± 12.08
	2 weeks after treatment	112.46 ± 8.82^ac^	127.46 ± 10.11^ac^	107.71 ± 8.01^ac^	3.17 ± 0.21^ac^	117.83 ± 9.07^ac^
Control group (*n* = 33)	Before treatment	135.04 ± 11.83	226.09 ± 11.87	170.98 ± 9.93	5.80 ± 0.69	160.11 ± 7.93
	2 weeks after treatment	119.08 ± 6.54^a^	131.87 ± 13.83^a^	114.82 ± 7.32^a^	3.67 ± 0.36^a^	121.03 ± 8.73^a^

Note

Before treatment, compared with the same group (^a^ *p* < .05); after treatment, compared with the control group (^b^ *p* < .05) and compared with the routine group (^c^ *p* < .05).

### 3.4 Comparison of cognitive function among the three groups

**FIGURE 4:**
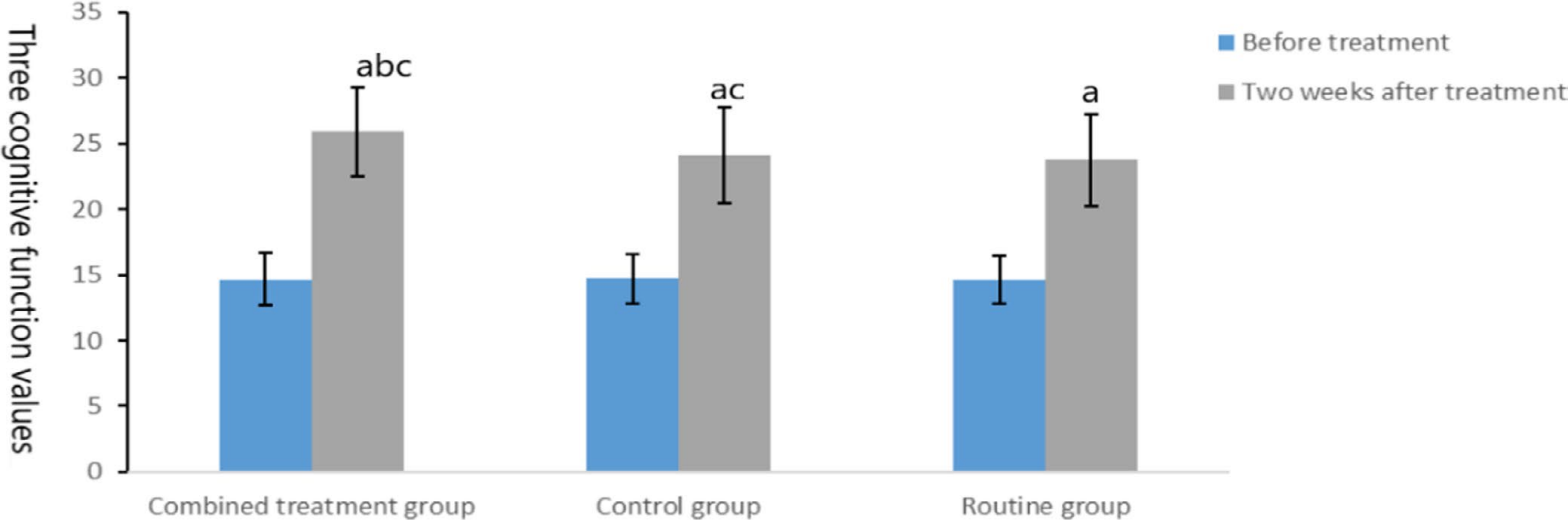
Comparison of cognitive function in three groups. The data of the combined treatment group, the control group, and the conventional group before treatment were 14.67 ± 1.99, 14.71 ± 1.88, and 14.61 ± 1.85, respectively. The data after 2 weeks treatment were 25.89 ± 3.42, 24.08 ± 3.67, and 23.76 ± 3.52, respectively. Before treatment, compared with the same group (^a^ *p* < .05); after treatment, compared with the control group (^b^ *p* < .05) and compared with the routine group (^c^ *p* < .05)

Before intervention, there was no significant difference in MoCA scores among the three groups (*t* = 0.023, *p* = .977), but after treatment, the scores of MoCA in the combined treatment group and the control group were better than those in the routine group, and the scores of MoCA in the combined treatment group were better than those in the control group (*t* = 2.073, *p* = .042), as shown in Figure 4.

### 3.5 Comparison of activities of daily living among the three groups

**FIGURE 5:**
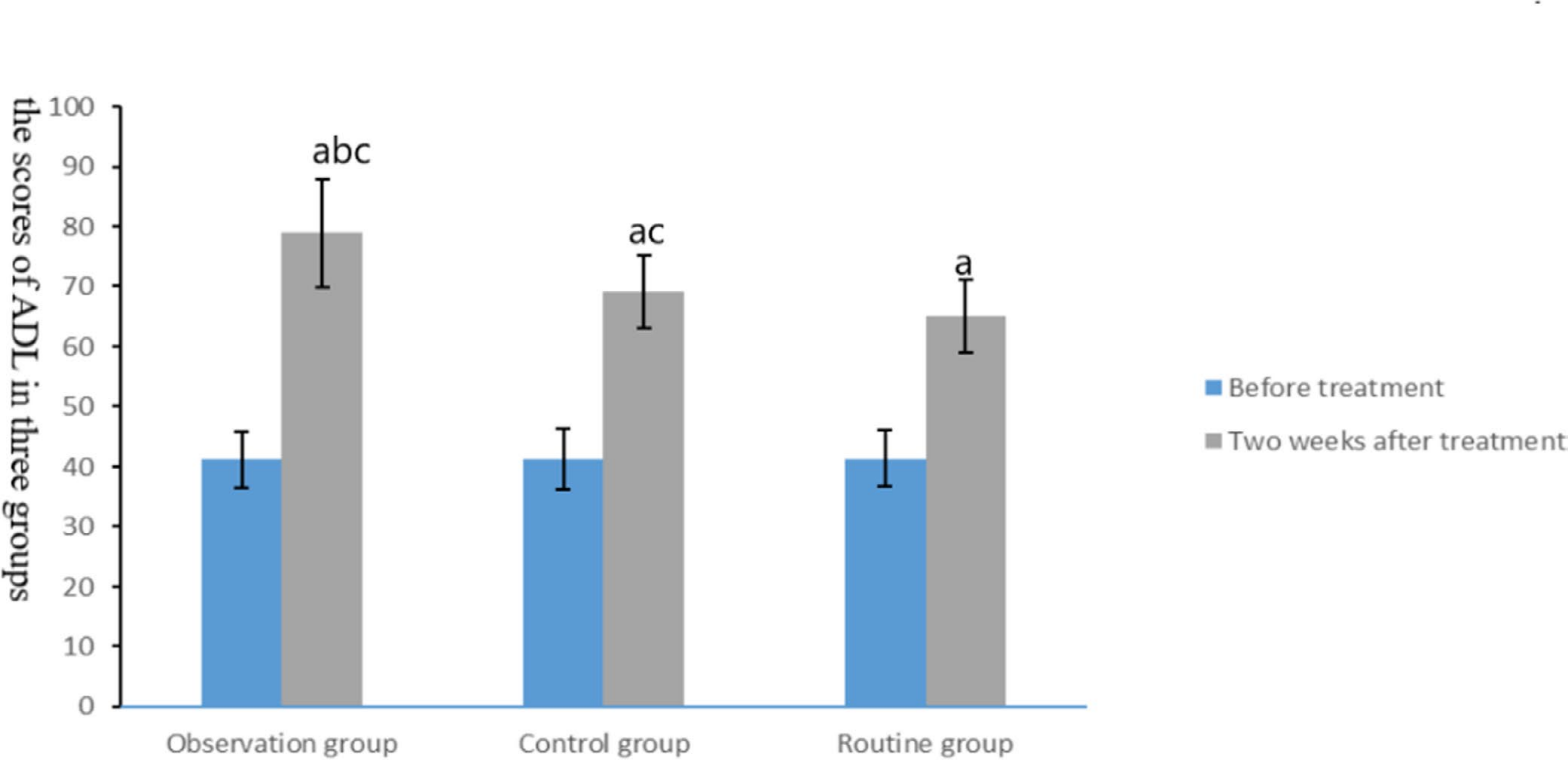
Comparison of activities of daily living in three groups. The data of the combined treatment group, the control group, and the conventional group before treatment were 41.18 ± 4.72, 41.21 ± 5.01, and 41.31 ± 4.68, respectively. The data after 2 weeks treatment were 78.93 ± 8.94, 69.08 ± 6.07, and 65.08 ± 5.98, respectively. Before treatment, compared with the same group (^a^ *p* < .05); after treatment, compared with the control group (^b^ *p* < .05) and compared with the routine group (^c^ *p* < .05)

Before intervention, no significant difference was observed in ADL scores among the three groups (*t* = 0.006, *p* = .994), but after treatment, the scores of ADL in the combined treatment group and the control group were better than those in the routine group, and the scores of ADL in the combined treatment group were better than those in the control group (*t* = 5.24, *p* < .01), as shown in Figure 5.

### 3.6 Comparison of adverse reactions among the three groups

There were no adverse reactions in the three groups. Detailed results are shown in Table 4. There was no significant difference in complications between the three groups (*p* > .05).

**TABLE 4:** Comparison of complications among three groups of patients

Type	Combined treatment group	Control group	Routine group	*p*
Rebleeding	1	2	2	>.05
Pulmonary infection	3	3	4	>.05
Gastrointestinal hemorrhage	1	1	1	>.05
Urinary tract infection	2	3	3	>.05

## 4 DISCUSSION

Acute intracerebral hemorrhage is one of the most common life‐threatening conditions in neurology, mainly caused by atherosclerosis, diabetes, and hypertension. The overall mortality of the disease can reach as high as 40% (Lai et al., 2019). Cerebral edema and cerebral hemorrhage are the main causes of death in patients with acute intracerebral hemorrhage. Cerebral hemorrhage can cause hematoma to oppress the surrounding normal tissue and lead to secondary brain injury. Brain edema is a type of secondary pathophysiological changes, which is highly associated with the abnormal release of inflammatory factors and blood decomposition products. It can result in the continuous increase of intracranial pressure, cause cerebral hernia, and aggravate the symptoms even death of patient (Chen, Xu, et al., 2019). Therefore, patients harboring early acute intracerebral hemorrhage should seek early and effective intervention. The main treatment principles include the reduction of blood pressure, hemostasis, and prevention of complications, in order to improve the prognosis of patients.

Oxiracetam is a new type of neurotrophic drug, which is a derivative of γ‐aminobutyric acid (GABA). Its molecular weight is small and can promote the binding of nucleic acid and protein, which is able to promote the combination of phosphatidylethanolamine and phosphatidylcholine in brain tissue. Oxiracetam can accelerate the energy metabolism of brain tissue and improve the utilization rate of glucose via the activation the glycolysis pathway. Additionally, it can improve cognitive function and activities of daily life through the acceleration of the metabolism of glutamate receptor, resulting in the increase of postsynaptic potential amplitude and the improvement of the functional reconstruction of brain tissue (Hu et al., 2017). Ginkgo biloba extract is extracted from ginkgo biloba leaves, mainly composed of ginkgo flavonoid glycosides, which can improve the cerebral and peripheral blood circulation (Ahlemeyer & Krieglstein, 2003). Ginkgo flavonoid glycoside is an effective free radical scavenger, which can directly play an antioxidant role, inhibit the formation of lipid peroxidation, and protect cell membrane from free radical damage. Ginkgo flavonoid glycosides can improve free radical metabolism, reduce lipid peroxidation, regulate and improve the activity of antioxidant enzymes, expand blood vessels, and reduce capillary permeability.

According to the study supported by Sun, Xu, and Zhang (2018), oxiracetam can reduce the volume of cerebral hematoma in patients with primary intracerebral hemorrhage. Based on the experiment on rats, Kaur, Chhabra, and Nehru (2013) confirmed that ginkgo biloba extract could promote the recovery of neuronal mitochondrial function, alleviate the energy metabolism of brain tissue, alleviate cerebral edema, and protect the neurological function after intracerebral hemorrhage. Ma and Wang (2019) conducted a study and confirmed that oxiracetam combined with ginkgo biloba extract can reduce cerebral edema and hematoma in patients with acute intracerebral hemorrhage. The results of this study showed that after treatment, the cerebral edema and hematoma in the combined treatment group and the control group were reduced and gained better results than those in the routine group, and the brain edema and hematoma in the combined treatment group were less than those in the control group, indicating that oxiracetam combined with ginkgo biloba extract can effectively reduce cerebral edema and cerebral hematoma, and was superior to oxiracetam alone and routine treatment. Based on the results of this study, after treatment, the mRS score and the case number of mRS > 3 in the combined treatment group and the control group were better than those in the routine group, and the mRS score and case number of mRS > 3 in the combined treatment group were better than those in the control group (*p* < .001), suggesting that oxiracetam combined with ginkgo biloba extract can effectively improve the recovery of neurological function. The abovementioned results may be linked to the fact that oxiracetam is able to regulate the excitability of nervous system and inhibit the transmission of neurohormones and neural signals by acting on neuroreceptors to improve neural function. Ginkgo biloba extract can increase the glucose supply and oxygen in the ischemic part of brain tissue, increase the number of neurotransmitter receptors, improve the neural state, and promote the recovery of neural function.

MMP‐9 is one of the main family members of matrix metalloproteinases, which participates in the decomposition of endothelial basement membrane (which can maintain the permeability of blood–cerebrospinal fluid barrier). If the content of MMP‐9 in the body increases, it can lead to endothelial basement membrane injury and the destruction of blood–cerebrospinal fluid barrier and the increase of tissue edema around cerebral hematoma. Hayman et al. found that MMP‐9 is a key factor in pathophysiology after SAH. In the study of patients and animal models, MMP‐9 caused brain edema after degradation of extracellular matrix protein and destruction of tight junctions (Hayman, Wessell, Gerzanich, Sheth, & Simard, 2017). Hence, MMP‐9 can be used in clinical diagnosis indicator of cerebrovascular and cardiovascular diseases (Borland et al., 2017). A variety of growth factors and cells in the body can regulate the secretion of MMP‐9, and TNF‐ α is an important inducing cytokine. Several clinical studies suggest that serum inflammatory factors such as IL‐6, TNF‐α, and hs‐CRP released after intracerebral hemorrhage are closely related to brain edema and brain injury (Csuka et al., 1999). TNF‐α is widely expressed, and its activity is multifaceted in intracranial injury, leading to endothelial permeability change, brain edema, leukocyte translocation, cell apoptosis, and cell necrosis (D'Mello, Le, & Swain, 2009). It has been proved that the level of IL‐6 in serum and the secretion of cytokines, including IL‐1 β and TNF‐α, increase with the increase of cerebrospinal fluid after brain injury (Dalla Libera et al., 2011). The results of the current study demonstrated that after treatment, the indexes of IL‐6, AQP‐4, MMP‐9, hs‐CRP, and TNF‐α in the combined treatment group and the control group were better than those in the routine group, and the indexes of CRP, PCT, and WBC in the combined treatment group were better than those in the control group (*p* < .05), indicating that oxiracetam combined with ginkgo biloba extract was able to effectively regulate the levels of inflammatory factors, AQP‐4, and MMP‐9 in serum. According to studies supported by Yao, Yao, Li, Nie, and Zhang (2016), oxiracetam can promote cerebral perfusion and improve the cognitive function of patients with intracerebral hemorrhage. Some studies (Herrschaft et al., 2012) have confirmed that ginkgo biloba extract can improve the activity of daily living in patients with hypertensive intracerebral hemorrhage. The results of this study showed that after treatment, the scores of MoCA and ADL in the combined treatment group and the control group were better than those in the routine group, and the scores of MoCA and ADL in the combined treatment group were better than those in the control group (all *p* < .05). Similar to the results of previous studies, our aggregated results suggested that oxiracetam combined with ginkgo biloba extract can effectively improve the cognitive function and daily living ability of patients. In the review of Geraghty, it is found that there are no successful drugs for the treatment of subarachnoid hemorrhage in clinic. The drugs in this study can be further studied (Geraghty, Davis, & Testai, 2019).

## 5 CONCLUSION

In the present study, significant differences were observed in the recovery of neurological function among the three treatment methods in patients harboring acute intracerebral hemorrhage. And the patients receiving oxiracetam combined with ginkgo biloba extract gained remarkable beneficial outcomes, but the outcomes may partly attribute to their natural recovery. Given the small sample size and grouping method included in our study, the deviation of the methodology may exist to cause the inaccuracy of results. Therefore, further studies with larger sample size are warranted to confirm the combined effect of oxiracetam and ginkgo biloba extract on the prognosis of patients with acute intracerebral hemorrhage.

## CONFLICT OF INTEREST

The authors declare there is no conflict of interest.

## AUTHOR CONTRIBUTION

Xiu‐Xiu Li and Shi‐Hui Liu contributed to the conception and design of the study. All authors participated in the clinical practice, including diagnosis, treatment, consultation, and follow‐up of patients. Su‐Jing Zhuang and Shi‐Feng Guo contributed to the acquisition of data. Shou‐Liang Pang and Shi‐Feng Guo contributed to the analysis of data. Xiu‐Xiu Li wrote the manuscript. Shi‐Hui Liu and Su‐Jing Zhuang revised the manuscript. All authors approved the final version of the manuscript.

## ETHICAL APPROVAL

The current study was approved by the hospital ethics committee, and all of patients signed the informed consent form.

## Data Availability

The datasets generated and analyzed during the current study are available from the corresponding author on reasonable request.
